# Potential risk of HIV transmission in barbering practice in Ethiopia: *from public health and microbiological perspectives*

**DOI:** 10.1186/1471-2458-12-707

**Published:** 2012-08-29

**Authors:** Fantahun Biadgelegn, Yeshambel Belyhun, Belay Anagaw, Desalegn Woldeyohannes, Feleke Moges, Asegedech Bekele, Andargachew Mulu

**Affiliations:** 1 Department of Medical Microbiology, Medical Faculty, Bahir Dar University, Bahir Dar, Ethiopia; 2 School of Biomedical and Laboratory Sciences, College of Medicine and Health Sciences, University of Gondar, P.O. Box 196, Gondar, Ethiopia; 3 Department of Anatomy, College of Medicine and Health Sciences, University of Gondar, P.O. Box 196, Gondar, Ethiopia; 4 Institute of Virology, Faculty of Medicine, University of Leipzig, Leipzig, Germany

## Abstract

**Background:**

HIV and other blood borne infections can be transmitted through the use of improperly sterilized and disinfected sharp equipments.

**Methods:**

A cross sectional study was conducted from January to June, 2010 to assess the potential risk of HIV transmission in barbering practice in Ethiopia from public health and microbiological perspectives. Barbers in barbershop were interviewed using pre-designed questionnaires and check lists were used to evaluate barbering practice. Microbiological data from tips of the sharpener before and after the barbering was collected and processed as per the standard procedure.

**Results:**

One hundred and twenty three barbering sessions and barbers were observed in which 106 (86.2%) were males. Ninety six (78%) of the respondents knew that HIV could be transmitted by sharing non-sterile sharp instruments. Among the total participants 59 (48%) had the correct knowledge of what sterilization mean and 111 (94.1%) of them believed its importance in their work place. Barbers had a mean knowledge score of 6 ± 1.5 out of a score of 10 regarding sterilization and disinfection as well as in the transmission of HIV in their work place. Three (2.5%) barbers were disagreed that unsterilized blade can transmit skin diseases and 26 (21.3%) of them believed disinfection is enough to avoid microbes from sharp objects. Ninety two (76.7%) barbers were using sterilization in their establishment. According to Likert scaling almost all sterilization and disinfection procedures were riskily practiced and respondents had poor level of knowledge. No significant association was found to influence the decontamination and sterilization of barbering equipments except monthly income, pre and post colony count of microbes identified. The isolation of normal skin flora in the pre-and post- sterilization and disinfectant procedures and less average percent colony reduction showed that sterilization and disinfectant practices in barbershop were generally poor that proofed proper sterilization and/or disinfection techniques were unfavorable.

**Conclusion:**

This study has revealed the presence of potential risk of HIV and other blood borne disease transmission among the barbers of the study areas. Thus continuous and intensified public health strategies on health education, training, supervision and monitoring are needed to facilitate the adoption of effective methods of sterilization and/or disinfection.

## Background

HIV and other blood borne pathogens can be transmitted from one person to another through the use of non-sterile needles, syringes, and other skin piercing equipment like blade and invasive instruments [1]. Proper sterilization of such instruments is therefore important to prevent HIV transmission. HIV is very sensitive to standardized methods of sterilization and disinfection and method designed to inactivate other virus such as hepatitis B virus [2,3].

Barbershop is one of the places where there is frequent use of some blades often without proper sterilization and the clients' face and skull skin can be scratched and may be cut by sharp equipments during shaving of their hair. In Ethiopia shared shaving equipment in barbershops are commonly practiced. Accidental scratch by sharp equipment in barbershops may create an opportunity for microorganisms, mainly HIV and other blood borne pathogens, to enter to the body easily and cause serious health problems to the clients [4]. In this case, it poses potential risks in the transmission of HIV because of the possibility of skin piercing injuries. Sterilization and/or disinfection in between shavings using sharp equipment will kill potential infectious agents and will prevent disease transmission among clients[4].

Heat is the most effective method for inactivating HIV and other blood borne pathogens. Methods for sterilization and high level disinfection based on heat are therefore the method of choice. High-level disinfection by boiling is feasible in most circumstances, as this requires only a source of heat, a container and water. In practical and field settings, high-level disinfection with chemicals is reliable. High level disinfectants which are effective for inactivating HIV are sodium hypochlorite with 0.1-0.5% chlorine, 2% chloramine, 70% ethanol, 70% 2-propanol, 2.5% poryvidone, 4% formaldehyde, 2% glutoral, 6% hydrogen peroxide [3,5].

In Ethiopia HIV/AIDS started spreading rapidly in 1980's after the notification of the first case in 1986 [5-7]. The prevalence of HIV infection in Ethiopia rapidly increased from 3.2% in 1993 to 10.63% in 2001 and now reduced to 2.1% [8-11] with 1.4 million peoples living with the virus. The main route of transmission is heterosexual intercourse. To mitigate the problem, the Ethiopian government has developed a national policy on HIV/AIDS in 1998. The policy is designed to guide the implementation of successful programs to prevent the spread of HIV/AIDS using the universal prevention and control methods including ensuring of safe use of sharp equipment like needles and razor blades [8,12]. However, the methods of sterilization and/or disinfection used by barbers in their work place are questionable throughout the country.

Although studies [13-16] appraising the knowledge, attitude, and practice about HIV transmission are conducted among various section of society, little attention has been given towards hair cutting as a place where such transmission occurs and consequently the study on the assessment of knowledge, attitude and practice about sterilization and/or disinfection of sharp equipment regarding HIV transmission was not well studied before. Hence the present study was conducted to assess the knowledge, attitude, and practice about sterilization of sharp equipment regarding HIV transmission in their work place; and to evaluate the microbiological efficacy of sterilization and/or disinfection methods used in barbershop.

## Methods

### Study design and settings

Across-sectional study was conducted among barbers and their barbershops in Gondar and Bahir Dar towns from January to June, 2010. Gondar and Bahir Dar cities are the major economical and administrative centers of Amhara Regional States of Ethiopia located 565 Km and 760 Km away from Addis Ababa, the nation capital, respectively and 180 Km far apart from each other. Bahir Dar and Gondar towns have an estimated total population of 256,999 and nearly 300, 000, respectively according to 2010 Central Statistical Authority of Ethiopia [17]. Barbering is one of income generating activities for many urban youths in many parts of Ethiopia including the study areas.

### Sampling and sample size determination

The source population for this study was all men barbershops of the Bahir Dar and Gondar cities. All men barbershops in these two cities were registered before the study and there were 267 barbershops. Considering 50% of the total coverage, 134 barbershops were selected for the study. The barbershops were stratified by geographic proximity and from each stratum barbershops were selected systematically using the sampling interval based on proportion. One barber was selected randomly for the interview in each barbershop if two or more barbers were available per a barbershop. Female barbershops and beauty salons, those unwilling to participate in the study, those who delivered incomplete data were excluded from the study.

### Data collection

Using pre-tested questionnaire and observational checklist data was collected. Prior to data collection, data collectors were oriented on the objective of the study, methods of interviewing and the importance of filling the format properly. The quality of the data was assessed and supervised during interviewing.

The pre-tested observation checklist used for data collection was designed to record the instruments used, type of decontamination, description of equipment decontamination procedures, names and certification of germicidal potency of decontaminant in terms of composition, concentration and expiration. Other practices observed included incidences of zero-cut hairstyles and accidental cuts as well as actions taken on client and instrument on such occasions. With the exception of the verification of decontaminants, other observations were done unobtrusively.

### Microbiological data

Sharpeners of the barbers who had been selected to the questionnaire were taken for this purpose. The ‘pre’ and ‘post’ sterilized and/or disinfected sharpeners tips were swabbed aseptically with a sterile moisten absorbent cotton swab and inoculated in to a separate sterile 1 ml normal saline labeled as “ pre” and “post’. The samples were delivered to Microbiology laboratory of Felege Hiwot Hospital and Gondar University Hospital, respective to each city within one hour in ice cold after collection. A known volume of samples from each tube were then inoculated to the Standard Plate Count Agar (SPCA which was prepared as per the instruction of the manufacturer labeled) by means of a sterile spreader [9].

Based on the standard microbiological procedures, plates were incubated aerobically for 20 hours at 35-37°C. Then plates were read for the presence or absence of colonies. Colonies were counted in each plate (the assumption that, each viable cell will form a colony is an indication of the members of viable cells in the original sample under study) [4]. Coagulase test was done to identify bacterial origins which are part of the normal flora of the skin which used as an indirect indicator whether other blood born pathogens were cleared after sterilization and/or disinfection procedures of barbers. According to the standard [6], colony count on average percentage reduction (APR) of each plate was calculated to determine the efficacy of the methods of sterilization and/or disinfection used in the barbers. Average percent colony reduction was calculated as number of colony on ‘pre’ plate minus number of colony count on ‘post’ plate divided by the number of colony on ‘pre’ plate multiplied by 100%.

### Data analysis

The collected raw data was entered to SPSS version 16-software package and analyzed based on variables set or the objective of the study. The score of one or zero was given based on the correct or wrong answer to individual knowledge questions and Likert scaling was used to measure the responses to each attitude statement [18]. The knowledge score of 75.0% or above was graded as good knowledge and below this cut-off point as poor knowledge. The score of 60.0% and below was graded as unfavorable attitude and above this cut-off point as favorable attitude. The presence of at least one risky practice was considered as poor while its absence as good practice [18]. The microbiological data (efficacy of methods of sterilization and/or disinfection) was analyzed by calculating the average percent reduction. Simple descriptive statistics was used to explain the findings of the study.

### Ethical consideration

Data collection was preceded after a written consent obtained from barbershop owners and an oral consent from the study participants of barbers. Ethical approval for the study was obtained from the Research and Publications Office of Bahir Dar University.

## Results

### Sociodemographic characteristics

One hundred twenty three (91.8%) barbers were participated during study period and the rest (8.2%) were excluded due to various reasons. Among the barbers participated in the study, 106 (86.2%) were males. The mean age was 24.6 ± 5.6 which ranges from 17 to 60 years old. Eighty four (71.8%) of the respondents had completed secondary school followed by 29 (24.8%) of participants who had completed the elementary school level. Majority 88 (71.5%) of the barbers had less than 5 years work experience as a barber and 29 (23.6%) of them had experience of 5–10 years.

### Practice of sterilization and disinfection techniques

Ninety seven (78.9%) of them had ever practiced sterilization. Ninety-two (76.7%) barbers were currently using sterilization in their barbershop and among these 82 (89.3%) of them used direct flame, 2 (2.2%) dry heat oven, 1 (1.1%) boiling. Out of the 117 (96.7%) barbers who used disinfectants currently, 49 (41.9%) used ethanol, 17 (14.5%) used berekina (bleach) and 44 (37.6%) were used no disinfectants. Eighty-eight (71.5%) of barbers didn’t know the concentration of disinfectants they used. Respondents were asked whether any program of inspection was carried out in their settings and 91 (89.2%) of them responded as there was no any programme of inspection by pertinent bodies like municipality, health center, AIDS club in their respective towns (Table 1). Almost all sterilization and disinfection procedures were riskily practiced according to Likert scaling.

**Table 1:** Assessment of sterilization and disinfection practices in male barbershops of Gondar and Bahir Dar cities, 2010

**Variables**	**Frequency N (%)**	**Likert scaling**
**Have you ever used sterilization? (n = 123)**		
Yes	97(78.9)	
No	26(21.1)	Risky practice
**Are you using sterilization now? (n = 120)**		
Yes	92(76.7)	
No	28(23.3)	Risky practice
**What types of sterilization do you use? (n = 92)**		
Autoclave	0(0)	
Dry heat oven	2(2.2)	
Boiling	1(1.1)	
Direct flame	82(89.3)	Good practice
Others	7(3.2)	
**Are you using disinfectant now? (n = 121)**		
Yes	117(96.7)	
No	4(3.3)	Risky practice
**What type(s) of disinfectant (= 117)**		
Ethanol	49(41.9)	Good practice
Phenol	3(2.6)	
Carboxylic acid	1(0.9)	
Berekina	17(14.5)	
Combination of disinfectants	3(2.6)	
No disinfectant at all	44(37.6)	
**Concentration of disinfectant used (n = 123)**		
96%	26(21.1)	
70%	9(7.3)	
Don’t know	88(71.5)	Risky practice
**Where do you get the disinfectants? (n = 117)**		
Pharmacy	48(39.7)	
Shops	57(47.1)	
Market	8(6.6)	Risky practice
Other (whole sale distributor)	4(3.3)	
**Is there any program of inspection carried out in your work area? n = (n = 102)**		
Yes	11(10.8)	
No	91(89.2)	Risky practice
**By whom inspection was carried out? (n = 9)**		
Municipality	3(2.4)	
Health center	4(3.3)	Good practice
AIDS club	0(0)	
Others	2(1.6)	
**How often the inspection was practiced? (n = 8)**		
Every week	0(0)	
Every month	2(1.6)	Risky practice
Every 3 months	1(0.8)	
Every 6 months	1(0.8)	
Every year	4(3.3)	

### Attitude of barbers towards sterilization and disinfection

About 72 (59.5%) of the respondents strongly agreed that sterilization of sharp instrument is necessarily in their work place for safe practices against the transmission of HIV and other diseases. Regarding disinfection practices, majority 66 (54.1%) of them believed or responded as ‘agree’ for the question ‘disinfection is enough in the barbers’ work place to protect their clients from HIV and other transmissible diseases’. Surprisingly, 3 (2.5%) of them ‘disagreed’ for the question ‘unsterile blade/equipment can transmit HIV and skin diseases’ (Table 2). Likert scaling showed that none of the respondents had favorable attitudes towards sterilization and disinfection importance and availability.

**Table 2:** Assessment of attitudes of barbers towards sterilization and disinfection in male barbershops of Gondar and Bahir Dar cities, 2010

**Variables**	**Frequency N (%)**	**Likert scaling**
**Sterilization of sharp instruments is necessarily found in your work place? (n = 121)**		
Strongly agree	72(59.5)	Unfavorable attitude
Agree	46(38.0)	
Disagree	2(1.7)	
Strongly disagree	1(0.8)	
**Disinfection is enough in your work place to avoid microbes from sharp objects (n = 122)**		
Strongly agree	30(24.6)	
Agree	66(54.1)	
Disagree	26(21.3)	Unfavorable attitude
Strongly disagree	0(0)	
**Unsterile blade can transmits HIV and skin diseases (n = 122)**		
Strongly agree	67(54.9)	Unfavorable attitude
Agree	52(42.6)	
Disagree	3(2.5)	
Strongly disagree	0(0)	

### Knowledge of barbers towards sterilization and disinfection and awareness of HIV transmission in their work place

Ninety six (78%) of the respondents knew that HIV and other bacterial and fungal skin infections could be transmitted by sharing non-sterile sharp barbershop equipments. Out of one hundred twenty three barbers, 59 (48%) had the correct knowledge of what sterilization mean and 111 (94.1%) of them also believed that it is important being practiced in their work place. All were able to mention at least one disease which could be transmitted by using unsterilized sharp objects. Almost all 119 (98.3%) knew sterilization is important in their work place (Table 3). However, their scored level of knowledge towards sterilization and disinfection as well as awareness in the transmission of HIV in their work place showed that 23 (18.7%) had excellent knowledge followed by 52 (42.3%) had good knowledge, 43 (35.0%) had fair knowledge, while the rest had poor 5 (4.1%) knowledge. All the barbers were aware of HIV/AIDS and they had a mean knowledge score of 6 ± 1.5 out of a maximum score of 10 regarding sterilization and disinfection as well as in the transmission of HIV in their work place (Table 3). However, using the Likert scaling, respondents’ had poor level of knowledge.

**Table 3:** Assessment of knowledge of barbers towards sterilization and disinfection as well as their awareness in the transmission of HIV in their work place of male barbershops of Gondar and Bahir Dar cities, 2010

**Variables**	**Frequency N (%)**	**Likert scaling**
**True about sterilization**		
Simply clean the surface of sharp object	31(25.2)	
Reducing microbial load on the surface of the object	13(10.6)	
Complete destruction of all form of microbes from an object	59(48.0)	Poor knowledge
All	18(14.6)	
No answer	1(0.8)	
**Do you know sterilization is important in your work place**		
Yes	111(94.1)	Good knowledge
No	7(5.9)	
**Do you know methods of sterilization?**		
Yes	119(98.3)	Good knowledge
No	2(1.6)	
**Which methods of sterilization do you know**		
Direct flame	100(92.6)	
Hot air oven	3(2.8)	
Boiling	4(3.7)	
Autoclave	1(0.9)	
**Do you think disinfection is sterilization**		
Yes	36(30.0)	
No	84(70.0)	Poor knowledge
**Disinfection is as effective as sterilization**		
Yes	65(67.0)	Poor knowledge
No	32(33.0)	
**If you think disinfection is not sterilization what are their difference.**		
No difference	48(39.3)	
Sterilization kill but not disinfectant	38(31.1)	Poor knowledge
Direct flame is better than sterilization	29(23.8)	
Sterilization reduce microbial load but not disinfectant	3(2.5)	
Sterilization gives partial protection	3(2.5)	
No knowledge	1(0.8)	
**Do you know any diseases that are caused by using unsterilized sharp equipment?**		
Yes	120(100)	Good knowledge
No	0(0)	
**Mention types of diseases, which could be transmitted by non sterilized sharp equipment's?**		
HIV/skin infections/fungus/dandruff, boil and others	96(78.0)	Poor knowledge
Skin infections / fungus and others but not mentioning HIV	27(22.0)	
**Scored level of Knowledge (Maximum of ten points)**		
Poor (1-3 points))	5(4.1)	
Fair (4-5 points)	43(35.0)	
Good (6-7 points)	52(42.3)	Good knowledge
Excellent (8-10 points)	23(18.7)	

### In-site observation of barbers practice on sterilization and disinfection

The check list observation of barbers’ practices showed that 112 (94.9%) and 113 (91.9%) of the barbers used at least one of sterilization and disinfection procedures, respectively. Ninety-eight (79.7%) of the barbers didn’t know the duration of time of contact when they used disinfect and/or sterilize their instruments. In 84 (80.0%) of barbers it was observed that they didn’t practice a correct sterilization and/or disinfection procedures. Almost all barbers had physical facilities including sheets and other common barber shop materials but none had sink, tap water, and first aid kits (Table 4).

**Table 4:** Assessment of sterility and decontamination status of barbering instruments during in site observation of barbers at their work place of male barbershops of Gondar and Bahir Dar cities, 2010

**Variables**	**Frequency N(%)**	**Likert scaling**
**Any sterilization procedures practiced**		
Yes	112(94.9)	
No	6 (5.1)	Risky practice
**Any disinfection procedure practiced**		
Yes	113(91.9)	Risky practice
No	10(8.1)	
**Name of chemicals and percentages available**		
Ethanol (96%)	7(5.9)	
Ethanol (70%)	53(44.5)	Good practice
Carboxylic acid	59(49.6)	
Berekina (Bleach)	0(0)	
Others	4(3.3)	
**Time of contact while disinfecting/sterilizing**		
1- 5 seconds	10(8.1)	
6-10 seconds	0(0)	
11-15 seconds	3(2.4)	
> 15 seconds	12(9.8)	
Don’t Know	98(79.7)	Risky practice
**Correct sterilization/ disinfection procedures followed**		
Yes	21(20)	
No	84(80)	Risky practice
**Assessment of physical facilities:**		
**● Sheets:**		
Yes	116(95.1)	
No	6(4.9)	
**● Common materials**		
Yes	123(100)	
No	0(0)	
**● Sink**		
Yes	0(0)	
No	123(100)	Risky practice
**● Tap water**		
Yes	0(0)	
No	123(100)	Risky practice
**● First aid kits**		
Yes	0(0)	
No	123(100)	Risky practice

### Microbiological data

Pre-procedural isolation showed that 15(12.5%) and 85(70.8%) were *Staphylococcus auerus* and Coagulase negative *Staphylococci* (CNS) species, respectively while post-procedural isolation showed 13 (10.8%) and 62(51.7%) of *S. aureus* and CNS, respectively to the pre and post procedures (Tables 5 and 6).

**Table 5:** The association of socio-demographic characteristics and practice of equipments decontamination among male barbershops of Gondar and Bahir Dar cities, 2010

**Characteristics**	**Decontamination practiced**		**Total**	**X**^**2**^	**P-value**
	**Appropriate**	**Inappropriate**			
**Sex**					
Male	98(92.5)	8(7.5)	106(100)	0.35	0.56
Female	15(91.9)	2(8.1)	17(100)		
Total	113(91.8)	10(8.1)	123(100)		
**Age**					
<20 years	10(100)	0(0)	10(100)	2.6	0.62
20-29 years	89(89.9)	10(10.1)	99(100)		
30-39 years	11(100)	0(0)	11(100)		
40-49 years	2(100)	0(0)	2(100)		
>50 years	1(100)	0(0)	1(100)		
Total	113(91.9)	10(8.1)	123(100)		
**Religion**					
Orthodox Christianity	105(93.8)	7(6.2)	112(100)	8.2	0.02
Muslim	4(80)	1(20)	5(100)		
Protestant	3(60)	2(40)	5(100)		
Total	112(91.8)	10(8.2)	122(100)		
**Educational status**					
No formal education	3(75)	1(25)	4(100)	1.49	0.47
Primary school	27(93.1)	2(6.9)	29(100)		
High school	77(91.7)	7(8.3)	84(100)		
College and above	0(0)	0(0)	0(0)		
Total	107(91.5)	10(8.5)	117(100)		
Monthly income in Birr*					
50-250	15(100)	0(0)	15(100)	6.92	0.22
251-500	43(93.5)	3(6.5)	46(100)		
501-750	15(83.3)	3(16.7)	18(100)		
751-1000	8(80)	2(20)	10(100)		
>1001	4(80)	1(20)	5(100)		
**Years of Services**					
<5 years	8(92.0)	7(8.0)	15(100)	0.73	0.95
5-10 years	26(89.7)	3(10.3)	29(100)		
11-15 years	4(100)	0(0)	4(100)		
16-20 years	1(100)	0(0)	0(0)		
>21 years	1(100)	0(0)	1(100)		
Total					
**Scored level of Knowledge (See Table**3**)**					
Poor	5(100)	0(0)	5(100)	3.2	0.37
Fair	38(88.4)	5(11.6)	43(100)		
Good	50(96.2)	2(3.8)	52(100)		
Excellent	20(87.0)	3(13.0)	23(100)		
Total	113(91.9)	10(8.1)	123(100)		
**Pre-organism isolated**					
No isolate	19(95.0)	1(5.0)	2(100)	0.48	0.79
*S.auerus*	14(93.3)	1(6.7)	15(100)		
CNS	77(90.6)	8(9.4)	85(100)		
Total	110(91.7)	10(8.3)	120(100)		
**Post-organism isolated**					
No isolate	41(91.0)	4(8.9)	45(100)	1.15	0.56
*S.auerus*	11(84.6)	2(15.4)	13(100)		
CNS	58(93.5)	4(6.5)	62(100)		
Total	110(91.7)	10(8.3)	120(100)		
**Pre-colony count**					
< 300	75(89.3)	9(10.7)	1(100)	3.51	0.74
301-600	21(100)	0(0)	1(100)		
601-900	8(88.9)	1(11.1)	9(100)		
901-1200	1(100)	0(0)	1(100)		
1201-1500	4(100)	0(0)	4(100)		
>1500	1(100)	0(0)	1(100)		
Total	110(90.7)	10(9.3)	108(100)		
**Post-colony count**					
< 300	98(90.7)	10(9.3)	108(100)	1.51	0.91
301-600	8(100)	0(0)	8(100)		
601-900	2(100)	0(0)	2(100)		
901-1200	1(100)	0(0)	1(100)		
1201-1500	1(100)	0(0)	1(100)		
>1500	0(0)	0(0)	0(0)		
Total	110(91.7)	10(8.3)	120(100)		
Average percent colony reduction					
1-20	5(100)	0(0)	5(100)	4.0	0.41
21-40	13(81.2)	3(18.8)	16(100)		
41-60	25(96.2)	1(3.8)	26(100)		
61-80	6(100)	0(0)	6(100)		
81-100	61(91.0)	6(9.0)	67(100)		
Total	110(91.7)	10(8.3)	120(100)		

* One US dollar = 14.5 Ethiopian Birr (at the time of data collection).

**Table 6:** The association of socio-demographic characteristics and practice of equipment sterilization practices among male barbershops of Gondar and Bahir Dar cities, 2010

**Characteristics**	**Sterilization practiced**		**Total**	**X**^**2**^	**P-value**
	**Appropriate**	**Inappropriate**			
**Sex**					
Male	97(95.1)	5(4.9)	102(100)	0.05	0.82
Female	15(93.8)	1(6.2)	16(100)		
**Age**					
<20 years	10(100)	0(0)	10(100)	1.61	0.81
20-29 years	88(93.6)	6(6.4)	94(100)		
30-39 years	11(100)	0(0)	11(100)		
40-49 years	2(100)	0(0)	2(100)		
>50 years	1(100)	0(0)	1(100)		
Total	112(94.9)	6(5.1)	118(100)		
**Religion**					
Orthodox Christianity	102(94.4)	6(5.6)	108(100)	0.53	0.77
Muslim	5(100)	0(0)	5(100)		
Protestant	4(100)	0(0)	4(100)		
Total	111(94.9)	6(5.1)	117(100)		
**Educational status**					
No formal education	4(100)	0(0)	4(100)	0.50	0.78
Primary school	27(96.4)	1(3.6)	28(100)		
High school	77(93.9)	5(6.1)	82(100)		
College and above	0(0)	0(0)	0(0)		
Total	108(94.7)	6(5.3)	114(100)		
**Monthly income**					
50-250	15(100)	0(0)	15(100)	19.4	0.002
251-500	43(100)	0(0)	43(100)		
501-750	16(100)	0(0)	16(100)		
751-1000	10(100)	0(0)	10(100)		
>1001	5(100)	0(0)	5(100)		
**Years of Practice**					
<5 years	80(94.1)	5(5.9)	85(100)	0.54	0.97
5-10 years	26(96.3)	1(3.7)	27(100)		
11-15 years	4(100)	0(0)	4(100)		
16-20 years	1(100)	0(0)	1(100)		
>21 years	1(100)	0(0)	1(100)		
Total	112(94.9)	6(5.1)	118(100)		
**Scored level of Knowledge (See Table**3**)**					
Poor	4(100)	0(0)	4(100)	1.96	0.58
Fair	38(92.7)	3(7.3)	4(100)		
Good	47(94.0)	3(6)	50(100)		
Excellent	23(100)	0(0)	23(100)		
Total	112(94.9)	6(5.11)	118(100)		
**Pre-organism isolated**					
No isolate	18(90.0)	2(100)	20(100)	3.28	0.19
*S.auerus*	14(100)	0(0)	14(100)		
CNS	79(97.5)	2(2.5)	81(100)		
Total	111(96.5)	4(3.5)	115(100)		
**Post-organism isolated**					
No isolate	42(95.5)	2(4.5)	44(100)	0.55	0.76
*S.auerus*	11(100)	0(0)	11(100)		
CNS	58(52.3)	2(3.3)	60(100)		
Total	111(96.5)	4(3.5)	115(100)		
**Pre-colony count**					
< 300	77(96.2)	3(3.8)	80(100)	25.9	0.00
301-600	20(100)	0(0)	20(100)		
601-900	8(88.9)	1(11.1)	9(100)		
901-1200	1(100)	0(0)	1(100)		
1201-1500	4(100)	0(0)	4(100)		
>1500	1(100)	0(0)	1(100)		
Total	111(96.5)	4(3.5)	115(100)		
**Post-colony count**					
< 300	100(97.1)	3(2.9)	103(100)	33.5	0.00
301-600	8(100)	0(0)	8(100)		
601-900	1(50)	1(50)	2(100)		
901-1200	1(100)	0(0)	1(100)		
1201-1500	1(33.3)	2(66.7)	3(100)		
>1500	1(100)	0(0)	1(100)		
Total	112(94.9)	6(5.1)	118(100)		
Average percent colony reduction					
1-20	4(80)	1(20)	5(100)	5.9	0.20
21-40	16(100)	0(0)	16(100)		
41-60	23(100)	0(0)	23(100)		
61-80	6(100)	0(0)	6(100)		
81-100	62(95.4)	3(4.6)	65(100)		
Total	110(91.7)	10(8.3)	115(100)		
Total	110(91.7)	10(8.3)	115(100)		

Average percent colony reduction of the above normal flora pathogens isolated were also determined before and after decontamination practices of the barbershop and only 67 (55.8%) showed 81-100% colony reduction followed by 26 (21.7%) and 16 (13.3%) which showed colony reduction from 41-60% and 21-40%, respectively. Similarly, the average percent colony reduction before and after sterilization practices showed that 65 (56.5%) of isolate showed 81-100% reduction which was followed by 23 (20%) and 16 (13.9%) of the isolate which showed 41-60% and 21-40% reduction, respectively (Tables 5 and 6).

No significant association was found to influence the decontamination and sterilization of barbering equipment except monthly income, pre and post colony count, respectively (Table 5 and 6).

## Discussion

There is a growing concern that barbering procedures could create opportunities for HIV as well as other blood borne and skin diseases transmission. In areas where such infections are common assessing knowledge, attitude and practice of barbers and to evaluate the efficacy of proper sterilization and/or disinfection techniques have a paramount importance for proper intervention. In Africa, barber shaving is probably one of a number of nonsexual cultural practices that may expose individuals to blood and blood-borne pathogens through the use of shared instruments repeatedly for different customers without intervening disinfection and sterilization and unaware of the concept of transmission of infectious agents [19]. For instance, the prevalence of saloon or roadside barber shaving has been reported to be as high as 34 - 49% in countries such as Ethiopia, Pakistan, and Bangladesh [20-22]. The practices done in barbershop, however, are largely underestimated and unaddressed as one of a route of blood-borne disease transmission [19,20,23]. In other countries, it was well reported that barbers used sharp instruments which may facilitate the transmission of HBV and HCV [15].

In the current study, the knowledge, attitude and practice of barbers and the efficacy of the sterilization and/or disinfection techniques practiced in the two cities of northwest Ethiopia was assessed for the first time. The majority of them had ever practiced sterilization by using the direct flame. The current most common practice was disinfection using ethanol although others like chlorine (sodium hypochlorate), phenolic compounds, quaternary ammonium compounds, iodine and iodophores inactivate organisms [24]. Using ethanol in the current study had not been a problem; however, most barbers didn’t know the concentration of the disinfectants and the appropriate place where quality types of disinfectants were purchased in the market. In similar study [14] in Nigeria, 52% of the disinfections involved the use of kerosene, a disinfectant not recommended for HIV inactivation; 48.3% of the disinfectants were not in the original containers while 53.4% of the sessions involved the use of same brush for cleaning clipper and brushing hair. Like the above Nigerian study, hand-held direct flame was a commonest (89.3%) sterilization technique in the current study but ultra-violet light sterilizer were not used in 50% of the sterilization process in Nigeria [14] and almost none in the current study. Though majority of the respondents were appeared to observe decontamination either as disinfection or sterilization, more than half of the disinfections were inappropriately done [25] which accounted 30% and 20% in the current study, respectively. This implies that the seemingly high disinfection rate among the respondents may only amount to a false sense of security to the clients and general public. This finding is similar to some previous studies on barbers’ practices [7,26].

Moreover, there was no organized body for inspection of barbering practices as well as there were no guidelines and governing rules and almost all sterilization and disinfection procedures were riskily practiced according to Likert scaling. These could show barbershop are the neglected areas of interest in regard to infection control and prevention strategies including HIV /AIDS in Ethiopia which is in agreement with previous study from Nigeria that indicated barbers’ activities could serve as a potential group for indirect transmission of HIV in the general population and such practices have not given any noticeable attention by the government and other bodies [14]. Another study also indicated that unlicensed activities by barbers are being done without knowing important health principles [27]. However, unlike under developing countries, activities of barbers are regulated in developed countries through a comprehensive training, licensing and monitoring programmes [24]. This is because the concept of universal precaution considers all blood and body fluids to be potentially infectious and all invasive instruments to be potentially contaminated if already used [28]. The responsibility to keep instruments free of infective agents lies on the barbers.

Our finding that none of the respondents had favorable attitudes towards sterilization and disinfection importance and availability was also consistent an Indian study [29] that showed barbers do not have any perception of unhealthy working practices in barbering and even awareness about threat of receiving hazardous infection from their customers is also unsatisfactory. Downey *et al.*[28] reported that creation of awareness among barbers about Hepatitis B, C and HIV would play a vital part in prevention and control of these infections. Barbers have low awareness about hepatitis and the risk of transmission of infectious agents by reuse of razors and scissors on multiple clients [15,30].

Many studies appraising the knowledge and practices about HIV transmission are conducted among various sections of society. However, little attention has been given towards and very few studies were reported about barber’s knowledge and practices regarding HIV transmission [13]. In the current study, 84.1% of the respondents knew that HIV and other skin infections could be transmitted by sharing non-sterile sharp barbershop instruments which was comparably to the study done in Nigeria [14]: However, it was contrary to a study from Nagpur, India [13] which had reported 81% were ignorant about modes of HIV transmission, particularly transmission via razor blades. Nevertheless, in the current study less than half of the participants (48%) had the correct knowledge of what sterilization mean despite the fact that all were able to mention at least one disease which could be transmitted by unsterilized sharp objects and almost all (98.3%) knew sterilization is important in their work place. Wazir *et al.*[29] in their study showed that the level of knowledge among barbers about health hazards associated with their profession was very poor. In line to this, in the current study all the barbers were aware of HIV/AIDS and they had a mean knowledge score of 6 ± 1.5 out of a maximum score of 10 regarding sterilization and disinfection as well as in the transmission of HIV in their work place. However, using the Likert scaling, respondents’ had poor level of knowledge. A study from Nigeria found a mean knowledge score of 7.2 out of a maximum score of 10 among barbers on HIV/AIDS [14]. Most barbers might knew about AIDS that had not resulted in any risk reduction practices [15] but practices of blade reuse have also been reported from a survey of barbers in India [13,15].

The observational check list of barbers’ practices showed that almost all barbers used at least one of sterilization and disinfection procedures but majority (80.0%) of barbers didn’t practice a correct sterilization and/or disinfection procedures and was consistent with the study in Nigeria [14]. Practices such as re-using the same blade, using fixed-blade razors, and performing inadequate disinfection procedures were common, especially among roadside barbers [8]. Specific HIV-risks of barbering procedures relating to HIV transmission have been documented in Nigeria and other African and Asian countries [13,31-33] that reported incidences of accidental cuts on scalps and poor hygiene practices, including low disinfection rates of re-useable instruments.

The concept of time of contact of sharp instruments during sterilization and disinfection practices, which if properly done can inactivate infectious agents including HIV was not considered in the majority of the barbers. Despite the availability and concentration of disinfectants used in the barbershop, time of contact of the materials and disinfectants showed only 9.8% of them practiced for more than 15 seconds and majority (79.7%) of the barbers didn’t know the duration of time of contact when they used disinfect and/or sterilize their instruments. Health and personal care workers are known to adhere strictly to decontamination guidelines for invasive instruments and the principle of ‘universal precautions’ [28,34] but regarding barbers such practices were uncommon and less practiced. Besides poor knowledge, practices and awareness, it is reported that barbers are paying more attention to the decoration, air conditioning, sound system, and availability of television in the shop, but they are not paying attention to risk factors associated with their profession in the prevention of diseases [13] which is consistent with our present study that none had sink, tap water and first aid kits.

The isolation of organism in the post-procedural sterilization and/or disinfection and low average percent colony reduction suggest that the sterilization and disinfectant procedures performed in barbershop were generally poor and indicates the knowledge, the attitudes, and practices of the barber on proper sterilization and/or disinfection techniques were unfavorable. This may result in the transmission of viruses and other pathogens. Reports elsewhere showed the likelihood of transmission increases with the frequency of reuse of razors and blades [20,35] under the above microbiological conditions. In particular, hands should be kept clean, gloves should be worn and adequate microbiological cleanliness of tools should be ensured. Inspection services should pay particular attention to whether the rules of handling used materials, a potential source of infection, are obeyed by workers [36].

Any of the sociodemographic characters and microbiological data was not found to influence the decontamination and sterilization of barbering equipments except monthly income and pre and post colony count which was associated to poor practices of sterilization and disinfection. This might be due the fact that the small number of study participants’. In opposite to this study, Chanda *et al.*[37] showed that barbers in the high-class peripheral areas were more likely to practice appropriate equipment decontamination than those from lower-class. Inappropriate practices may be due to lack of practical knowledge about decontamination and potency of disinfectants. In another study even the barbers working in shops in high-class areas had less than secondary level education and most barbers started their practice at a very young age of 10–12 years which limits their knowledge about transmission of diseases from the instruments used [16]. A barber’s profession is closely linked to the beliefs of an individual [27] and traditional and low-paid barbers in many developing countries earn their livelihood by providing shaving and hairdressing services in the marketplace and on the street side [15,19]. Such similar findings showed that although almost all socio-demographic variables were not associated to the practices of barbering in the current study, they could play pivotal role for poor or good practices.

## Conclusions

This study has revealed the presence of potential risk of HIV and other blood borne disease transmission among the barbers of the study area which was evidenced by the use unfavorable sterilization and disinfection procedures as well as poor knowledge, attitude and practices regarding sterilization and/or disinfection and HIV transmission. For effective control of transmission HIV, other blood born pathogens and skin infections from invasive barber instruments in Ethiopia, a comprehensive and intensified public health approach has to be adopted with the involvement of all relevant sectors and barbers should not be neglected. Thus, we recommended that attention should be given to hygienic and universal precautions practices in barbershops through routine supervision and monitoring by pertinent bodies. Practical-oriented health education, training and supervision and monitoring should be organized for the barbers on equipment decontamination with emphasis on the use of correct procedure of sterilization and disinfection.

## Competing interests

The authors declare that they have no competing interests.

## Authors’ contributions

AM and FM: Conceived, designed and proposed the research idea. FM: involved in consecutive proposal development and in grant searching and securing. BA, AB, FB, and YB: involved in data collection. DW and YB: involved in data entry, clearance, analysis, and interpretation of the findings. YB: responsible for drafting the manuscript. All authors involved in reviewing the manuscript and approval for publication.

## Pre-publication history

The pre-publication history for this paper can be accessed here:

http://www.biomedcentral.com/1471-2458/12/707/prepub

## References

[B1] WHO/UNAIDS AIDS Epidemic Update 2006 World Health Organization, Geneva

[B2] Plog BA Fundamentals of industrial hygiene 1996 4 National Safety Council, Chicago,IL 429

[B3] WHO Guidelines on sterilization and high-level disinfection method effective against HIV/ AIDS action WHO reports 1988 Global program on AIDS, Geneva

[B4] Monica C Laboratory practice in Tropical countries 2000 Cambridge University Press, Cambridge 49 50

[B5] Croser D Chipping J Cross-infection control in general dental practice: a practical guide for the whole dental team 1989 Quintessence Pub. Co, cop, London

[B6] Wistreich G Microbiology laboratory fundamental Applications 1997 EastLos Angeles College, Prenticehal

[B7] Salami KK Titiloye MA Brieger WR Otusanya SA Observations of barbers’ activities in Oyo State, Nigeria: Implications for HIV/AIDS transmission Int’l Quarterly of Community Health Education 2006 24 4 319 330 17695087

[B8] MOH AIDS in Ethiopia; disease prevention and control 2000 3 MOH, Addis Ababa Ethiopia

[B9] MOH Federal Ministry of Health of Ethiopia, AIDS in Ethiopia 2006 6 MOH, Addis Ababa

[B10] USAID/Ethiopia PEPFAR midterm evaluation of the integrated management of adolescent and adult illness project 2009 The Global Health Technical Assistance Project, 1250 Eye St., NW, Suite 1100 Washington, DC 2005

[B11] Shabir J Fasile HG Derege L Knowledge, attitude, and practice on high risk factors pertaining to HIV/AIDS in rural community Ethiop Med J 1995 33 1 1 6 7895741

[B12] Central Statistical Authority and ORC Macro Ethiopia Demographic and Health Survey 2000. Central Statistical Authority 2001 Addis Ababa and ORC Macro, Calverton, Maryland

[B13] Khandiat DW Ambadekar NN Vasudeo ND Knowledge and Practice about HIV transmission among barbers of Nagpur city Indian J Med Sci 1999 53 167 171 10695229

[B14] Arulogun OS Adesoro MO Potential risk of HIV transmission in barbering practice among professional barbers in Ibadan, Nigeria Afr Health Sci 2009 9 1 19 25 20842238 PMC2932524

[B15] Janjua NZ Nizamy MAM Knowledge and Practices of Barbers about Hepatitis B and C Transmission in Rawalpindi and Islamabad JPMA 2004 54 116 15129868

[B16] Waheed Y Saeed U Safi SZ Chaudhry WN Qadri I Awareness and risk factors associated with barbers in transmission of hepatitis B and C from Pakistani population: barber’s role in viral transmission Asian Biomed 2010 4 3 435 442

[B17] Central Statistical Authority of Ethiopia The 2010 Population and Housing Census of Ethiopia 2011 Central Statistical Authority, Addis Ababa Ethiopia

[B18] Daniel J Mueller A Handbook for researchers and practitioners Measuring social attitude 1986 New York College presses 8 17

[B19] Khaliq AA Smego RA Barber shaving and blood-borne disease transmission in developing countries S Afr Med J 2005 95 2 94 96 15751200

[B20] Bari A Akhtar S Rahbar MH Luby SP Risk factor for hepatitis C virus infection in male adults in Rawalpindi-Islamabad, Pakistan Trop Med Int Health 2001 6 732 738 10.1046/j.1365-3156.2001.00779.x 11555441

[B21] Kefenie H Desta B Mengesha S Zewide D Kebede T Prevalence of HIV-1 antibodies in patients with sexually-transmitted disease Ethiop Med J 1991 29 63 69 2060508

[B22] Khan LA Chowdhury MZ Begum RA Sexually-transmitted diseases among immigrants seeking jobs abroad J Prev Soc Med 1999 18 41 45 12179654

[B23] Daily Times (2007): 10 barbers diagnosed with HIV/AIDS, 52 with hepatitis in three years http://server.Kbriislamaba.go.id/index.php?option=com_content&task=view&id=336&Itemid=43.Accessed 20 May 2011

[B24] Anonymoussterilization 2006 2006 at http://www.lience.sate.tx.us/barbers/barberfaq.htm. Retrieved Martch 3, 2007

[B25] Awodele OO Emeka PM Agbamuche NC Akintonwa A The anti-microbial activities of some commonly used disinfectants Afr J Biotechnol 2007 6 8 987 990

[B26] Isaac WE Lawali M Lawali M Evaluation of HIV occupational risk amongst traditional barbers and itinerant nail cutters in North-West Nigeria Int Conf AIDS 2004 D10381 Abstract No

[B27] Al Aboud D Al Aboud K Ramesh V Barbers’ Education; a Window to Improve the Public about Skin Diseases J Ayub Med Coll Abbottabad 2007 19 1 66 17867487

[B28] Downey C Can Salons spread infections? Third Age Health Encyclopedia 2005 2005. Retrieved 10/7/2011 from http://www.getincontrol.org/isinews.htm#

[B29] Wazir MS Mehmood S Ahmed A Jadoon HR Awareness among barbers about health hazards associated with their profession J Ayub Med Coll Abbottabad 2008 20 2 35 38 19385454

[B30] Hardy DB Cultural practices contributing to the transmission of human immunodeficiency virus in Africa Rev Infect Dis 1987 9 1109 1119.37 10.1093/clinids/9.6.1109 3321361

[B31] Zewudie T Legesse W Kurkura G Knowledge, attitudes and Practices among barbers in South-western Ethiopia Africa Newsletter on Occupational Health and safety 2002 1269 1271

[B32] Ibrahim MT Opara WE Tanimowo T Knowledge of HIV/AIDS, infection prevention practices and accidental skin cuts in barbing saloons in Sokoto, Nigeria Nigeria Medical Practitioner 2007 51 6 123 127

[B33] Jeffers E History timeline of the barbering 2002 Retrieved on 14/10/2007 from http://www.edjeffersbarbermuseum.com/index.html

[B34] Katner HP Buckley RL Smith MU Henderson AM Endoscopic cleaning and disinfection procedure for preventing iatrogenic spread of human immunodeficiency virus J Fam Pract 1988 27 3 271 276 3418300

[B35] El-Sadawy M Ragab H el-Toukhy H Hepatitis C virus infection at Sharkia Governorate, Egypt: seroprevalence and associated risk factors J Egypt Soc Parasitol 2004 34 367 384 15124747

[B36] Bilski B Marynowicz Zdrowotnej KP Akademia MIM Marcinkowskiego K Pozna A Knowledge, hygiene behavior and risk of blood borne infections in the selected staff of beauty parlors and hairdressing salons Med Pr 2006 57 6 517 524 17533988

[B37] Chanda SK Khan KH Sharing of razor-blade in salons and risks of spreading HIV in Bangladesh The 3rd IAS Conference on HIV pathogenesis and Treatment 2004 Abstract No WePe 10.5p02

